# *Helicobacter suis* binding to carbohydrates on human and porcine gastric mucins and glycolipids occurs via two modes

**DOI:** 10.1080/21505594.2018.1460979

**Published:** 2018-05-15

**Authors:** Médea Padra, Barbara Adamczyk, John Benktander, Bram Flahou, Emma C. Skoog, János Tamás Padra, Annemieke Smet, Chunsheng Jin, Richard Ducatelle, Tore Samuelsson, Freddy Haesebrouck, Niclas G. Karlsson, Susann Teneberg, Sara K. Lindén

**Affiliations:** a Department of Medical Biochemistry and Cell Biology, Institute of Biomedicine, Sahlgrenska Academy, University of Gothenburg, Gothenburg, Sweden; b Department of Pathology, Bacteriology and Avian Diseases, Ghent University, Belgium; c Laboratorium of Experimental Medicine and Pediatrics, Faculty of Medicine and Health Sciences, University of Antwerp, Antwerp

**Keywords:** Helicobacter suis, Helicobacter pylori, bacterial adhesion, host–pathogen interactions, mucin, glycosylation, glycolipid

## Abstract

*Helicobacter suis* colonizes the stomach of most pigs and is the most prevalent non-*Helicobacter pylori* Helicobacter species found in the human stomach. In the human host, *H. suis* contributes to the development of chronic gastritis, peptic ulcer disease and MALT lymphoma, whereas in pigs it is associated with gastritis, decreased growth and ulcers. Here, we demonstrate that the level of *H. pylori* and *H. suis* binding to human and pig gastric mucins varies between individuals with species dependent specificity. The binding optimum of *H. pylori* is at neutral pH whereas that of *H. suis* has an acidic pH optimum, and the mucins that *H. pylori* bind to are different than those that *H. suis* bind to. Mass spectrometric analysis of mucin *O*-glycans from the porcine mucin showed that individual variation in binding is reflected by a difference in glycosylation; of 109 oligosaccharide structures identified, only 14 were present in all examined samples. *H. suis* binding to mucins correlated with glycans containing sulfate, sialic acid and terminal galactose. Among the glycolipids present in pig stomach, binding to lactotetraosylceramide (Galβ3GlcNAcβ3Galβ4Glcβ1Cer) was identified, and adhesion to Galβ3GlcNAcβ3Galβ4Glc at both acidic and neutral pH was confirmed using other glycoconjugates. Together with that *H. suis* bound to DNA (used as a proxy for acidic charge), we conclude that *H. suis* has two binding modes: one to glycans terminating with Galβ3GlcNAc, and one to negatively charged structures. Identification of the glycan structures *H. suis* interacts with can contribute to development of therapeutic strategies alternative to antibiotics.

## Introduction

*Helicobacter suis* is a Gram negative, spiral shaped bacterium which colonizes the stomach of 60–95% of pigs at slaughter age and adult sows and boars [1–3]. *H. suis* colonization in the pig stomach is associated with chronic gastritis, decreased daily weight gain [4] and ulcers in the *pars oesophagea* (non-glandular stomach) [1,3]. *H. suis* is the most prevalent non-*Helicobacter pylori Helicobacter* species found in the human stomach, [5] signifying its zoonotic importance. In the human host, *H. suis* has been reported to contribute to the development of chronic gastritis, [6] peptic ulcer disease [7] and gastric mucosa-associated lymphoid tissue (MALT) lymphoma [8].

The first barrier that gastric pathogens encounter is the mucus layer, of which the main components are highly glycosylated mucin glycoproteins [9]. In a healthy human stomach, MUC5AC and MUC6 are the main secreted mucins [10], however, in gastric disorders, expression of MUC5B and MUC2 has also been described [11]. MUC5AC is a major constituent of the surface mucous gel layer, whereas the expression of MUC6 is limited to the glands [12]. In the pig stomach the mucins produced by the surface epithelium differ from the gland mucins in apoprotein content and length of the highly glycosylated domains [13]. These mucins might represent the porcine equivalents of the human MUC5AC and MUC6 mucins, but their identity has not yet been defined. Mucins carry a multitude of branched oligosaccharide chains attached to serine and threonine residues [14]. The high diversity of mucosal oligosaccharide chains in the gastrointestinal tract forms an extensive repertoire of potential binding sites for bacteria [15–17].

*Helicobacter pylori* is the most common human gastric pathogen, colonizing half of the world's population [18]. The majority of *H. pylori* reside in the gastric mucus layer where they can bind to the gastric mucins [19]. The most thoroughly investigated *H. pylori* adhesins are the blood group antigen binding adhesin (BabA) that binds to Lewis b (Le^b^) and H-type1 structures [20] and the sialic acid binding adhesin (SabA) that binds *N*-acetyllactosamine-based gangliosides with terminal α3-linked NeuAc, with a preferential binding to sialyl-Le^x^ (SLe^x^) and sialyl-Le^a^ (SLe^a^) [21,22]. Other outer membrane proteins (OMPs), e.g. LabA, AlpA and AlpB, may also serve as adhesins [23]. Across the gastric mucus layer, there is a pH gradient ranging from acidic in the lumen to neutral at the epithelial surface. Mucins from healthy humans carry mainly neutral glycans, and bind to BabA positive *H. pylori* strains in a Le^b^/H-type1 dependent manner and with a neutral pH optimum [19,24]. Mucins from humans with gastric inflammation or infection carry sialylated structures including SLe^a/x^. Binding to these mucins occurs via two mechanisms: via SabA with an apparent neutral pH optimum, and via a charge dependent mechanism at acidic pH [19,24]. *H. suis* genome analyses revealed that *H. suis* lacks homologs of several *H. pylori* adhesion factors, including BabA, SabA, and the adherence-associated lipoproteins AlpA and AlpB [25]. Conversely, *H. suis* contains some OMPs similar to the major OMP families described in *H. pylori,* which may be involved in binding to the gastric mucosa, [25] such as HorB [26] and the *H. pylori* adhesin A (HpaA) which was initially described as a sialic acid binding adhesin, but later identified as a lipoprotein [27].

The aims of the present study were to identify and further characterize the pig gastric mucins, investigate if *H. suis* binds to pig and human gastric mucins and glycolipids and, if so, define binding-active structures. We demonstrated that binding occurs to both human and pig mucins and glycolipids. Furthermore, *H. suis* binding occurs via two modes of adhesion: to Galβ3GlcNAcβ3Galβ4Glcβ1 at both neutral and acidic pH, and to negatively charged structures at acidic pH.

## Results

### H. *suis* resides in the mucus layer above the epithelial cells, but can also be found in the lamina propria and associated with parietal cells

**Figure 1.**
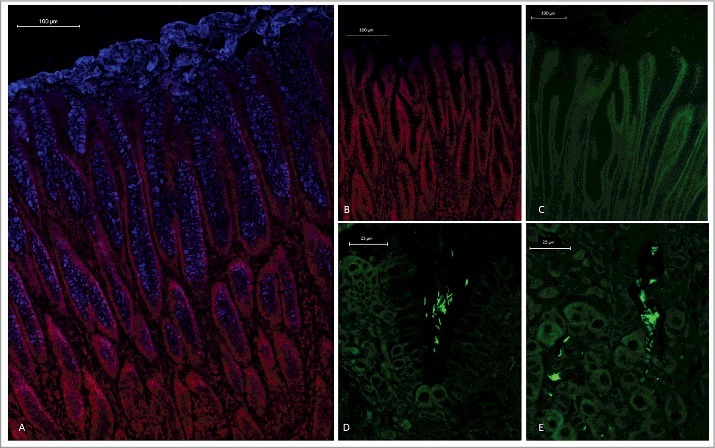
Spatial distribution of MUC5AC and *H. suis* in porcine gastric specimens. A. Porcine gastric tissue fixed with Carnoy´s fixative to retain the mucus layer was stained with a fluorescently labelled antibody against MUC5AC (blue), demonstrating a thick mucus layer above the epithelium. Nuclei and cytoplasm were stained with HCS CellMask™ (red). B. Negative control for MUC5AC staining (i.e. no MUC5AC antibody was added) with nuclei and cytoplasm outlined with HCS CellMask™ (red). C, D, E. Fluorescent *in situ* hybridization using an *H. suis* specific probe on *H. suis* –free (C) and infected (D, E) porcine gastric tissue sections. In these images, mucus is not visible, but the tissue is outlined by dull green auto fluorescence. *H. suis* (bright green color) was detected in the mucus layer in the gastric pits (D). Of the *H. suis* present in the tissue, most were found in the lamina propria and/or associated with the (fried egg shaped) parietal cells (E).

An antibody that recognizes isolated pig MUC5AC, [28] stained foveolar epithelial cells as well as the thick secreted mucus layer on pig gastric tissue sections (Figure 1A), similarly to the distribution previously shown for MUC5AC in the human stomach [29]. Fluorescence *in situ* hybridization on gastric tissue sections from *H. suis* infected pigs demonstrated the presence of *H. suis* in the mucus layer lining the surface epithelium and the gastric pits (Figure 1D), similarly to that *H. pylori* resides in the mucus layer above the epithelial cells [19,20]. In line with previously published electron micrographs of *H. suis* inside canaliculi of the acid producing parietal cell, [30,31] we also detected *H. suis* close to parietal cells, although most of the *H. suis* in the tissue were present in the intercellular space/lamina propria (Figure 1E).

### Characterization of mucins from pigs that were not infected with H. suis

**Figure 2.**
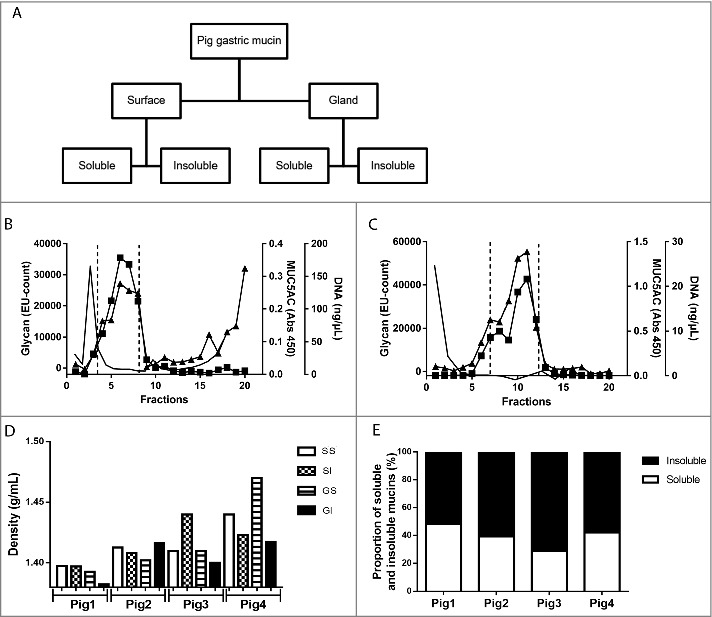
Isolation, solubility and density of porcine gastric mucins. A. Each specimen was separated into four types of mucin samples based on their tissue location and solubility in GuHCl. Mucins were then isolated from these samples using two steps of isopycnic CsCl density-gradient centrifugation. B. Fractions from the first density-gradient centrifugation (4 M GuHCl/1.39 g/mL) were analyzed for carbohydrate (▴), MUC5AC (▪), and DNA (─). The vertical dashed lines indicate how the mucin containing fractions were pooled for further purification in the second density-gradient centrifugation. C. Fractions from the second density-gradient centrifugation (0.5 M GuHCl/1.50 g/mL) were analyzed for carbohydrate (▴), MUC5AC (▪), and DNA (─). The vertical dashed lines indicate how the mucin containing fractions were pooled for further experiments. D. The density of the pig gastric mucins ranged from 1.38 to 1.47 g/mL. E. The proportion of mucins that were insoluble in GuHCl, was higher than the soluble (Mann-Whitney U-test: p ≤ 0.05). Abbreviations: SS: surface soluble, SI: surface insoluble, GS: gland soluble, GI: gland insoluble.

To identify the interaction between the porcine mucins and the pathogen during the initial colonization, mucins were isolated from the antral region of the gastric mucosa of pigs determined to be free of gastric *H. suis* using PCR and fluorescent in situ hybridization. During the extraction of mucins from the tissue, surface mucus was separated from the deeper gland material and mucins were further divided into soluble and insoluble groups based on their solubility in guanidinium chloride (GuHCl) resulting in four sample groups for each pig (Figure 2A). The mucins were purified from other proteins and DNA using two subsequent isopycnic density gradient centrifugation steps in 4 and 0.5 M GuHCl, due to that some samples had some overlap between the mucin containing fractions and the DNA containing fractions after the first centrifugation (Figure 2B and C). Mucins, localized by the overlapping peaks of carbohydrate reactivity and antibody reactivity against the MUC5AC mucin (Figure 2B and C), were present as a peak with a maximum at the density between 1.38 and 1.47 g/mL (1.41 ± 0.005, mean ± SEM, Figure 2D). Distinct differences in density were neither identified between mucins isolated from the surface epithelium or glands, nor between soluble and insoluble mucins, although the mucin density differed somewhat between pigs. Quantification of the carbohydrate content under the mucin peaks showed that insoluble mucins were slightly more predominant than soluble mucins in all pigs; the proportion of insoluble mucins ranged from 52% to 71% (60.4 ± 8.1, mean ± SEM, Figure 2E). After the second density gradient centrifugation, the mucin containing fractions were again pooled to provide four samples (surface soluble (SS), surface insoluble (SI), gland soluble (GI) and gland insoluble (GI)) from each individual for further experiments.

### Confirmation of presence of MUC5AC in the porcine stomach

In the healthy human stomach, MUC5AC, MUC6 and MUC1 are expressed, and MUC2 and MUC5B can also appear during disease [32]. Using antibodies against these human mucins, we identified immunoreactivity against MUC5AC in the porcine gastric surface epithelium (Figure 1A) and against isolated porcine gastric mucins (Figure 2B and C). In line with the histochemical results, mass spectrometry analysis identified porcine MUC5AC in six out of seven analyzed mucin samples (Supplementary table 2). A total of 18 peptides were identified via LC-MS[2] providing 16.8% coverage of the partially sequenced porcine MUC5AC (entry pig_muc5acTSallignment http://www.medkem.gu.se/mucinbiology/databases/index.html). Porcine MUC6 (entry gi545805660) was also detected by MS analysis but only in two out of seven samples analyzed and only two matching peptides were found with 1.2% protein sequence coverage. Porcine MUC5B (entry pig_muc5bTSallignmentAK238178.1_1_ORF1) was detected in six out of seven samples with six peptides (coverage 7.5%). Porcine MUC2 was not found in any of the samples, however, peptides homologous with human MUC2 (MJ080907, coverage 1.3%) was detected in one out of seven samples, thus, we can not exclude the possibility that small amounts of porcine MUC2 may be present.

The proteomic results support that MUC5AC is present in pig stomachs, and indicate that MUC6 and MUC5B also may contribute to the mucin repertoire of a healthy porcine stomach.

### *H. suis* binding to porcine mucins had an acidic pH optimum and differed between individuals and mucin types

**Figure 3.**
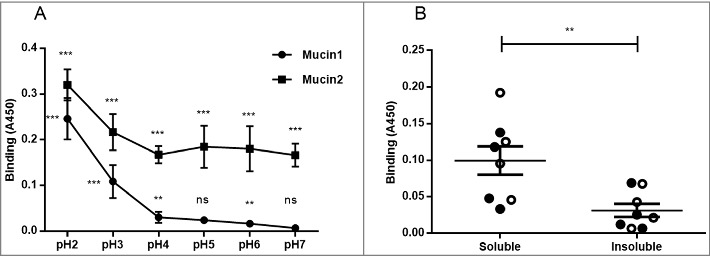
Effect of pH and mucin type on *H. suis* binding to pig gastric mucins. A. pH dependence of *H. suis* binding to mucins. Results are shown as mean ± SEM of bacterial binding after subtraction of background signal at each pH. ** and *** indicate p ≤ 0.01 and 0.001, respectively, in an unpaired two sided t-test comparing the binding to the negative control background at each pH. This assay was performed on four mucin samples, with similar results, ranging between the pronounced pH dependence shown with mucin 1 to the flatter curve seen with mucin 2. B. Effect of mucin type (surface (•), gland (○), soluble and insoluble) on *H. suis* binding at pH 2. Results are expressed as medians with interquartile ranges. ** indicates p ≤ 0.01 (Mann-Whitney U-test).

We analyzed *H. suis* binding to gastric mucins isolated from four pigs at pH 2–7, i.e. the pH range present in the pig stomach. *H. suis* bound to mucins from all four pigs at all pH tested (p ≤ 0.01). Binding was highest at pH 2 and gradually decreased towards neutral pH, though the gradient differed between mucins, with some samples retaining binding also at neutral pH whereas for other samples binding was only detected at acidic pH (Figure 3A). These results indicate that there may be two types of interactions between *H. suis* and mucins: one binding mode that is dependent on acidic pH and one that is functional also at neutral pH.

### H. suis and H. pylori binding to human gastric mucins displayed opposite pH dependency and differed in specificity

**Figure 4.**
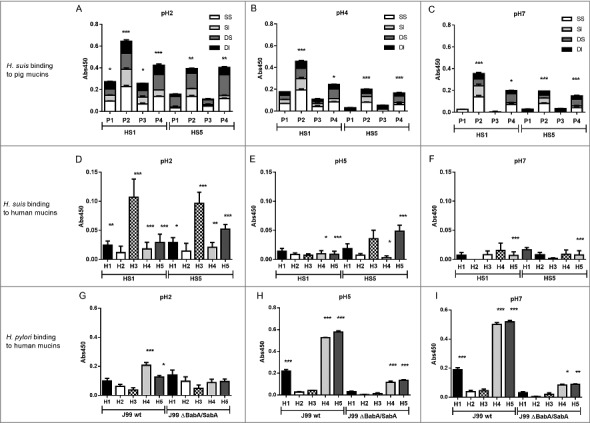
*H. suis* binding to pig and human gastric mucins. A, B, C. *H. suis* strains HS1 and HS5 binding to pig mucin groups (SS – surface soluble, SI – surface insoluble, GS – gland soluble, GI – gland insoluble) isolated from the stomach of four pigs (P1-4). D, E, F. Binding of *H. suis* strains HS1 and HS5 to human gastric mucins isolated from five human patients (H1-H5). G, H, I. Binding of *H. pylori* strain J99 wild type and its isogenic *ΔbabA/ΔsabA* deletion mutant to human gastric mucins. Results are shown as mean ± SEM of bacterial binding after subtraction of background signal at each pH. *, ** and *** indicate p ≤ 0.05, 0.01 and 0.001, respectively, One Way ANOVA, Dunnett´s post hoc test.

We selected pH 2, 4 and 7 to examine the binding of two strains (*H. suis* HS1 and HS5) to 16 pig gastric mucin samples purified from four *H. suis* negative pigs (P1-P4). The *H. suis* mucin binding differed between individuals (p ≤ 0.001, One-way ANOVA, Figure 4A-C), indicating binding to a specific glycan structure/group of structures via adhesin(s) as glycosylation differs between individuals. Furthermore, *H. suis* binding to GuHCl soluble mucins was more pronounced than to insoluble ones (p ≤ 0.01, Figure 3B) while no difference was detected between mucins derived from gland or surface mucosa (p = 0.798, Figure 3B).

Since *H. suis* is a zoonotic pathogen, we also investigated binding to human mucins (H1-H5), selected based on their previously described differences in *H. pylori* binding ability and glycosylation [19,24,33]. Similarly to the binding pattern obtained with porcine mucins, *H. suis* binding to human mucin samples differed between the five samples investigated, and the most pronounced binding occurred at pH 2 and to sample H3 (Figure 4D-F). As the main difference between the mucins is in glycosylation, this indicates that binding occurs to glycans. The binding specificity of *H. suis* differed from that of *H. pylori* both with regards to that *H. pylori* adhesion to mucins had a neutral pH optimum, and that the mucins that *H. pylori* bound to came from other individuals and were differently glycosylated than the ones *H. suis* bound to (Pearson r: -0.05, compare Figure 4D-F with Figure 4G-I). For *H. pylori*, the differential binding pattern between the wild type J99 strain and its isogenic *babA* and *sabA* adhesin deletion mutant, identified adhesin specific binding to human samples 1, 4 and 5 at pH 5 and 7 (Figure 4G-I), and for sample 4 and 5 some residual adhesin specific binding remained also at pH 2. Binding with the *H. pylori* adhesin deletion mutant at pH 2 was low and did not differ between individual mucins (Figure 4G-I). Thus, that *H. suis* binding differed between samples, together with the results above, indicate that similarly to what previously has been described for *H. pylori* [19,24,34], *H. suis* binding occurs to specific glycan structure(s) present on mucins, although the structure specificity and pH dependency differs from that of *H. pylori*.

**Figure 5.**
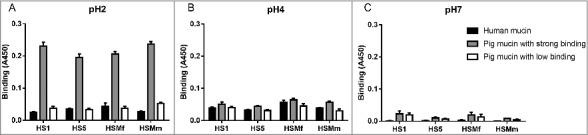
Comparison of binding of four *H. suis* strains to pig and human gastric mucins. *H. suis* binding at pH 2 (A), pH 4 (B) and pH 7 (C) to a human mucin, a pig mucin with high *H. suis* binding ability and a pig mucin with low *H. suis* binding ability.

To investigate if the binding pattern of the *H. suis* strains HS1 and HS5 was representative for more *H. suis* strains, we analyzed binding of another two strains (HSMf and HSMm) to mucins that were chosen to display a broad range of different glycans: a human mucin, a pig mucin with high *H. suis* binding ability and a pig mucin with low *H. suis* binding ability: all four strains had similar binding specificities and pH preferences (Figure 5).

### Porcine and human gastric mucin glycosylation

**Figure 6.**
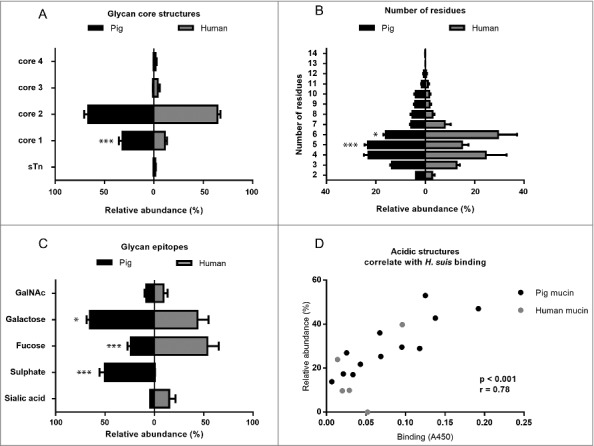
Porcine and human mucin *O*-glycosylation. A. Relative abundance of pig and human structures containing core 1–4 and sialyl-Tn. B. Size distribution (number of carbohydrate residues/glycan) of pig and human mucin *O*-glycans. C. Relative abundance of terminal glycan residues and sulfation on pig (n = 3, 12 subfractions) and human (n = 5) mucin glycans. Stars indicate statistically significant difference between pig and human mucin glycans, *, ** and *** indicate p ≤ 0.05, 0.01 and 0.001, respectively, Two-way ANOVA. D. Scatter plot of the amplitude of *H. suis* binding to mucins at pH 2 against their relative abundance of acidic glycan structures among pig (mainly sulfated structures, black) and human (mainly sialylated structures, grey). The r in the figures denotes the Pearson correlation coefficient, pooled for the human and pig data (pig mucins only: r = 0.85, human samples alone are too few to perform correlation analysis on).

To elucidate the structures responsible for binding and differences in binding between samples, we analyzed the carbohydrate structures present on the mucins using mass spectrometry. Mass spectrometric analysis of released mucin *O*-glycans was performed on all four mucin fractions from three of the four pigs: pig 3 was excluded from this analysis as histology showed signs of inflammation even though no infection was detected. We identified in total 109 oligosaccharide structures in the examined porcine mucin samples (43-93 structures from each sample and 56–104 from each individual, supplementary table 1). Only 14 of these were present in all of the examined samples, which indicate high inter-individual variation among porcine gastric mucin glycan structures. The oligosaccharides were mainly extended core 1 (mean ± SEM, 31.8 ± 3.7%) and core 2 glycans (mean ± SEM, 66.5 ± 4.0%) while the relative abundances of extended core 3 and core 4 structures, as well as the sialyl-Tn antigen (NeuAcα2,6GalNAcol) were below 1% (Figure 6A). The length of *O*-glycans varied between 2 and 14 residues (Figure 6B), and the glycans on GuHCl soluble mucins were shorter than those on insoluble mucins. In addition, five human mucin samples were selected for this study, based on their high variation in mucin glycosylation, thereby providing a wide range of potential structures for bacterial binding [17]. We identified 176 *O*-linked oligosaccharides (40-95 from each individual). The characterized structures were mainly extended core 2 *O*-glycans (64 ± 2%), although structures with core 1, 3 and 4 were also detected (Figure 6A). The relative abundance of extended core 1 *O*-glycans was higher in pig than human mucins (p < 0.001, Two-way ANOVA). Only 14 of the 176 oligosaccharides were present in all the five human samples. The glycan chain lengths ranged from 2 to 12 residues (Figure 6B). Since the terminal residues usually are vital parts of the binding epitopes, we quantified the relative abundance of these: galactose was the most common terminal residue among pig glycans, whereas fucose was most abundant among human mucins (Figure 6C). Both human and pig mucins had a low degree of sialylation (mean ± SEM, pig: 4.3 ± 1.1%, human 16.0 ± 6.8%). However, on 50% of the terminal pig glycan structures a sulfate group was present (mean ± SEM, 50.25 ± 5.1%), a modification that was virtually absent among the human samples (0.6 ± 0.2%, Figure 6C). The relative abundance of mucin glycans with terminal galactose or sulfate were higher in pigs than in humans (p < 0.001, p < 0.05, respectively, Two-way ANOVA), whereas the relative abundance of fucose terminating glycans were higher in human mucins (p < 0.001, Two-way ANOVA).

### Statistical correlations between *H. suis* binding and mucin glycans

**Figure 7.**
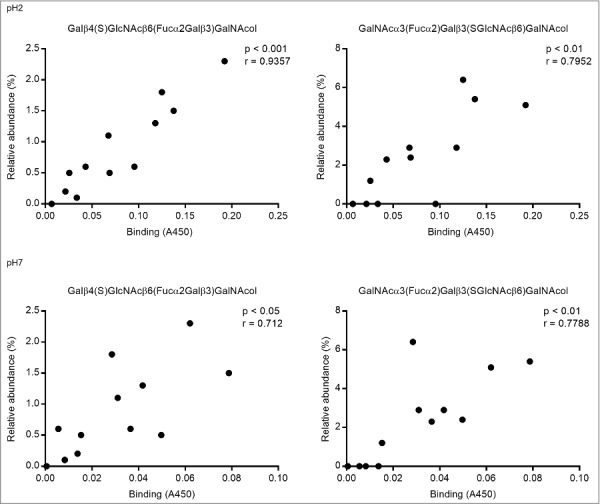
Scatter plots between *H. suis* adhesion and the relative abundance of the two structures that were associated with binding at both pH 2 and 7. The r in the figures denotes the Pearson correlation coefficient.

**Figure 8.**
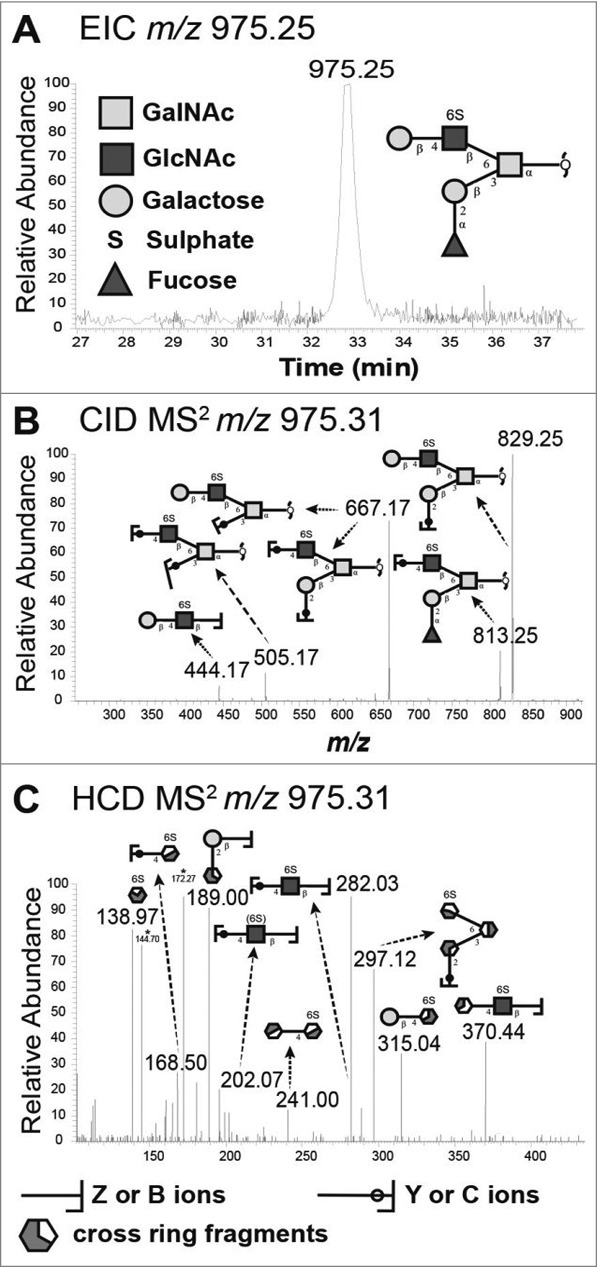
Identification of the *m/z* 975.25 structure (Galβ4(6S)GlcNAcβ6(Fucα2Galβ3)GalNAcol) from porcine gastric mucin *O*-glycans by LC-MS/MS. A. Extracted ion chromatogram (EIC) of *m/z* 975.25 [M-H]^−^ B. Collision-induced dissociation (CID)-based MS/MS fragmentation spectra with annotated fragments C. Higher-energy collisional dissociation (HCD)-based MS/MS low mass region (*m/z* 110–390) showing fragments specific for blood group H on C3 arm and a type 2 LacNAc on C6 with 6-linked sulfate on GlcNAc. * denotes background ions. The Consortium for Functional Glycomics cartoons were used to represent *O*-linked glycan structures.

We analyzed the relation between the bacterial binding amplitude and glycan structures: among the terminal residues, the relative abundance of sulfated as well as total acidic (i.e. sulfated and/or sialylated) glycans detected correlated well with the level of *H. suis* binding at pH 2 (Pearson r_10_ = 0.85, p ≤ 0.001, Figure 6D). The maximum *H. suis* binding to any individual human mucin was less than half compared to the binding to porcine mucins. The human glycans carried low amounts of sulfate (0 – 1%), however, the sample with the most pronounced binding was the sample that had the highest relative abundance of sialic acid (Figure 6D, Table 1), indicating that one *H. suis* mucin binding mode may be charge dependent. Of the 109 glycan structures present on the porcine gastric mucin samples, the abundance of seven glycans correlated with the *H. suis* binding level at pH 2, whereof two were also associated with binding at pH 7 (Table 2 and Figure 7). The two structures the abundance of which was associated with binding at both neutral and acidic pH were Galβ4(S)GlcNAcβ6(Fucα2Galβ3)GalNAcol and GalNAcα3(Fucα2)Galβ3(SGlcNAcβ6)GalNAcol (Figure 7). The abundance of Galβ4(S)GlcNAcβ6(Fucα2Galβ3)GalNAcol glycan structure correlated the best with binding at pH 2 (r = 0.94, p ≤ 0.001, structure identification shown in Figure 8).

**Table 1.** Relative abundance of human mucin glycan structures that correlated with *H. suis* binding in pig mucins (Table 2). Abundance of human mucin glycan motifs implicated to potentially be of relevance to *H. suis* binding from the statistical correlations between binding and relative abundance on pig mucins (Table 2). Although the number of samples was too low to perform correlation studies, sialylated and sulfated structures terminating in Gal and overall sialylated and sulfated structures were highest in sample H3, coinciding with that this sample had the highest *H. suis* binding ability (Figure 4).

	Relative abundance (%)				
Carbohydrate epitope	H1	H2	H3	H4	H5
Acidic structures (sialylated and/or sulfated)	10	10	40	24	0
Gal terminating structures (including blood group B)	8	72	30	49	60
Gal terminating acidic structures	2	4	12	9	0
GalNAc terminating structure (blood group A and LacdiNAc)	1	4	1	19	21
Fucα2Galβ3 (ABH blood group antigens)	60	7	33	28	27

**Table 2.** Porcine mucin glycan structures whose relative abundance correlate with the amplitude of *H. suis* binding to the mucins at pH 2 (A) or pH 7 (B). The two structures that had statistical correlation (p < 0.05) at both pH 2 and pH 7 are indicated with *. There were no positive statistical associations with the remainder of the identified glycan structures (listed in supplementary table S1).

				
		Correlation with binding		
Mass (*m/z*)	Structure	p value	Pearson r	Relative abundance (%)
A				
667	6SGlcNAcβ1,6(Galβ3)GalNAcol	p ≤ 0.01	0.7371	2 – 7
813	SGlcNAcβ6(Fucα2Galβ3)GalNAcol	p ≤ 0.05	0.6963	2 – 17
870	GlcNAcα4(S)Galβ3(GlcNAcβ6)GalNAcol	p ≤ 0.01	0.7111	0 – 5
975	Galβ4(S)GlcNAcβ6(Fucα2Galβ3)GalNAcol*	p ≤ 0.001	0.9357	0 – 2
1016	GalNAcα3(Fucα2)Galβ3(SGlcNAcβ6)GalNAcol*	p ≤ 0.01	0.7952	0 – 6
1121	Fucα2Galβ4(S)GlcNAcβ6(Fucα2Galβ3)GalNAcol	p ≤ 0.01	0.749	0 – 6
1381	GalNAcα3(Fucα2)GalGlcNAcβ3Galβ3(SGlcNAcβ6)GalNAcol	p ≤ 0.05	0.6778	0 – 1
B				
733	GalNAcα3(Fucα2)Galβ3GalNAcol	p ≤ 0.01	0.7849	0 – 2
975	Galβ4(S)GlcNAcβ6(Fucα2Galβ3)GalNAcol*	p ≤ 0.05	0.712	0 – 2
1016	GalNAcα3(Fucα2)Galβ3(SGlcNAcβ6)GalNAcol*	p ≤ 0.01	0.7788	0 – 6

### *H. suis* binds to Galβ3GlcNAcβ3Galβ4Glcβ1Cer glycolipid present in pig stomach

**Figure 9.**
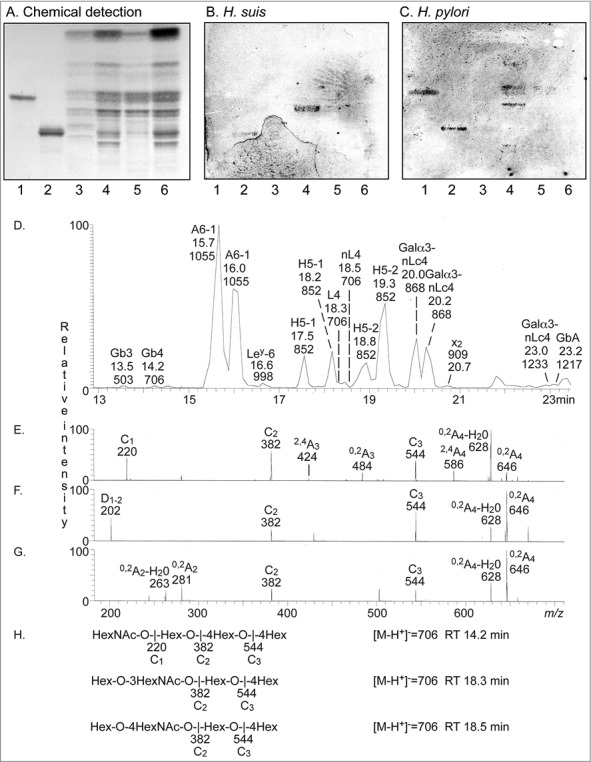
Characterization of *H. suis* binding glycosphingolipids from pig stomach. A. Thin-layer chromatogram after detection with anisaldehyde B. Autoradiogram obtained by binding of ^35^S-labeled *H. suis* (HS5) at pH 7.2. C. Autoradiogram obtained by binding of ^35^S-labeled *H. pylori* strain J99 wt at pH 7.2. The glycosphingolipids were separated on aluminum-backed silica gel plates, using chloroform/methanol/water 60:35:8 (by volume) as solvent system. Lanes 1–4 were total non-acid glycosphingolipids isolated from the stomach of four individual pigs, 40 µg/lane. D-G. LC-ESI/MS of the oligosaccharides obtained by digestion of the non-acid glycosphingolipid fraction of pig stomach (shown in chart A, lane 2) with *Rhodococcus* endoglycoceramidase II. D. Base peak chromatogram from LC-ESI/MS of the oligosaccharides derived from the non-acid glycosphingolipid fraction of pig stomach. E. MS^2^ spectrum of the ion at *m/z* 706 (retention time 14.2 min). F. MS^2^ spectrum of the ion at *m/z* 706 (retention time 18.3 min). G. MS^2^ spectrum of the ion at *m/z* 706 (retention time 18.5 min). H. Interpretation formulas showing the deduced oligosaccharide structures. Gb3, Galα4Galβ4Glc; Gb4, GalNAcβ3Galα4Galβ4Glc; A6-1, GalNAcα3(Fucα2)Galβ3GlcNAcβ3Galβ4Glc; Le^y^-6, Fucα2Galβ4(Fucα3)GlcNAcβ3Galβ4Glc; H5-1, Fucα2Galβ3GlcNAcβ3Galβ4Glc; L4, Galβ3GlcNAcβ3Galβ4Glc; nL4, Galβ4GlcNAcβ3Galβ4Glc; H5-2, Fucα2Galβ4GlcNAcβ3Galβ4Glc; Galα3-nLc4, Galα3Galβ4GlcNAcβ3Galβ4Glc; x_2_, GalNAcβ3Galβ4GlcNAcβ3Galβ4Glc; Galα3-nLc4, Galα3Galβ4GlcNAcβ3Galβ4GlcNAcβ3Galβ4Glc; GbA, GalNAcα3(Fucα2Galβ3GalNAcβ3Galα4Galβ4Glc.

To identify the binding specificity of *H. suis*, we examined binding of ^35^S-labeled bacteria to glycosphingolipids isolated from porcine stomach (Figure 9). Here, a binding to a compound migrating in the tetraosylceramide region was obtained (Figure 9B, lane 2). The *H. suis* binding glycosphingolipid was also recognized by *H. pylori* (Figure 9C, lane 2).

To further characterize the pig gastric glycosphingolipids, the non-acid glycosphingolipid fractions from the four pigs were hydrolyzed with *Rhodococcus* endoglycoceramidase II, and the oligosaccharides obtained were analyzed by LC-ESI/MS using a graphitized carbon column [35]. LC-ESI/MS of oligosaccharides, using graphitized carbon columns, gives resolution of isomeric saccharides, and the carbohydrate sequence can be deduced from series of C-type fragment ions obtained by MS^2^. In addition, diagnostic cross-ring ^0,2^A-type fragment ions are present in MS^2^ spectra of oligosaccharides with a Hex or HexNAc substituted at C-4, and thus allow differentiation of linkage positions.

The base peak chromatogram obtained from LC-ESI/MS of the oligosaccharides obtained by hydrolysis of the *H. suis* binding non-acid fraction from pig 2 (shown in Figure 9A and B, lane 2) is shown in Figure 9D. Molecular ions corresponding to oligosaccharides ranging from trisaccharides (detected as [M-H^+^]^−^ ions at *m/z* 503) to heptasaccharides (were detected as [M-H^+^]^−^ ions at *m/z* 1217 and 1233) were found. All molecular ions were subjected to MS^2^, and the oligosaccharides thereby identified are given in the legend of Figure 9. Molecular ions at *m/z* 706 correspond to an oligosaccharide with one HexNAc and three Hex. Here, three sets of peaks at *m/z* 706 were present, eluting at 14.2 min, 18.3 min and 18.5 min, respectively. The MS^2^ spectrum of the ion at *m/z* 706 at retention time 14.2 min (Figure 9E) had a C-type fragment ion series (C_1_ at *m/z* 220, C_2_ at *m/z* 382, and C_3_ at *m/z* 544), demonstrating a HexNAc-Hex-Hex-Hex sequence. The ^0,2^A_3_ fragment ion at *m/z* 484 demonstrated a 4-substituted Hex. Thus, a globotetra saccharide (GalNAcβ3Galα4Galβ4Glc) was tentatively identified. MS^2^ of the ion at *m/z* 706 at the retention time 18.7 min (Figure 9F) allowed a preliminary identification of a lactotetra saccharide (Galβ3GlcNAcβ3Galβ4Glc). This was concluded from the C-type fragment ions (C_2_ at *m/z* 382 and C_3_ at *m/z* 544) identifying a Hex-HexNAc-Hex-Hex sequence. This MS^2^ spectrum had a prominent D_1-2_ ion at *m/z* 202, which is obtained by a C_2_-Z_2_ double cleavage and diagnostic for a 3-substituted HexNAc [36]. The MS^2^ spectrum also lacked a ^0,2^A_2_ fragment ion at *m/z* 281, further demonstrating that the HexNAc was substituted at 3-position. Thus, LC-ESI/MS verified the presence of lactotetraosylceramide in the *H. suis* binding glycosphingolipid fraction. The MS^2^ spectrum of the ion at *m/z* 706 at the retention time 18.5 min (Figure 9G) was different. This spectrum had a prominent ^0,2^A_2_ fragment ion at *m/z* 281, and a ^0,2^A_2_-H_2_O fragment ion- at *m/z* 263, demonstrating a terminal Hex-HexNAc sequence with a 4-substitution of the HexNAc, *i.e*. a type 2 chain. In combination with the C_2_ ion at *m/z* 382 and the C_3_ ion at *m/z* 544, this demonstrated a neolactotetra saccharide (Galβ4GlcNAcβ3Galβ4Glc). The three MS^2^ spectra all had ^0,2^A_4_ ions at *m/z* 646, and ^0,2^A_4_-H_2_O ions at *m/z* 628, which were derived from cross-ring cleavage of the 4-substituted Glc of the lactose unit at the reducing end.

In subsequent binding assays using pure and structurally characterized reference glycosphingolipids a binding of *H. suis* to lactotetraosylceramide (Galβ3GlcNAcβ3Galβ4Glcβ1Cer) was observed, whereas lactotriaosylceramide (GlcNAcβ3Galβ4Glcβ1Cer), neolactohexaosylceramide (Galβ4GlcNAcβ3Galβ4GlcNAcβ3Galβ4Glcβ1Cer) and globotetraosylceramide (GalNAcβ3Galα4Galβ4Glcβ1Cer) were non-binding (data not shown). There was no binding of *H. suis* to the acid glycosphingolipids (sulfatide and gangliosides) of porcine stomach (data not shown). It should here be noted that due to too low incorporation of radioactive label into the fastidious *H. suis*, the results from the glycosphingolipid binding assays were difficult to reproduce, and in many cases no signal was obtained. Therefore we next investigated if *H. suis* bound to Lacto-N-tetraose (LNT, Galβ3GlcNAcβ3Galβ4Glc) conjugated to human serum albumin (HSA) using the same microtiter based assay that we used for the mucin binding analysis.

### *H. suis* binds to a synthetic Galβ3GlcNAcβ3Galβ4Glc conjugate at both acidic and neutral pH and at acidic pH to DNA (proxy for acidic charge)

**Figure 10.**
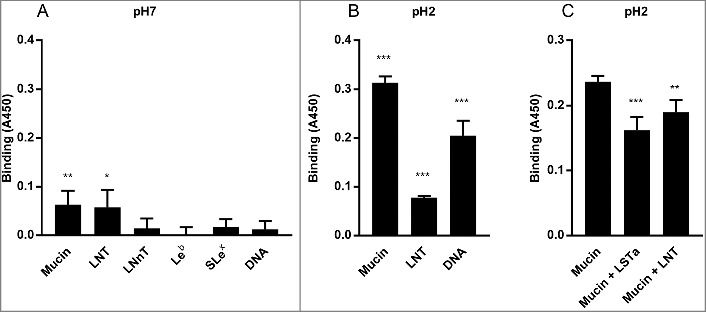
*H. suis* binding to synthetic glycoconjugates and inhibition of *H. suis* mucin binding. A. *H. suis* (HS1) binding to pig gastric mucin, LNT, LNnT, Le^b^, Sle^x^ (glycans conjugated to HSA) and DNA at pH 7. B. *H. suis* binding to pig gastric mucin, LNT conjugated to HSA and DNA (used as a marker for acidic charge without structural resemblance of mucins) at pH 2. C. Binding of *H. suis* to mucins was inhibited by pre-treatment of the bacteria with LNT and LSTa. To confirm the specificity of the inhibition, the experiment was also performed with monosaccharides that are part of the structures that inhibited binding (glucose, galactose, *N*-acetyl-glucosamine) and also with unrelated monosaccharides (fucose, *N*-acetyl-galactosamine). None of these structures interfered with the binding of the bacteria to the mucin (data not shown). Data are shown after subtracting background control for each pH. * indicates *p* ≤ 0.05, ** *p *≤ 0.01 and *** *p* ≤ 0.001, One-way ANOVA, Dunnett´s post hoc test.

In line with the glycolipid binding results, *H. suis* bound to LNT conjugated to HSA at pH 7 (p ≤ 0.05, Figure 10A) and in line with that *H. suis* did not bind to neolactohexaosylceramide (Galβ4GlcNAcβ3Galβ4GlcNAcβ3Galβ4Glcβ1Cer), no binding was detected to HSA conjugated Lacto-N-neotetraose (*LNnT*, Galβ4GlcNAcβ3Galβ4Glc). No binding was detected to the *H. pylori* binding structures Le^b^ and SLe^x^ conjugated to the same carrier either (Figure 10A). The *H. suis* LNT binding ability remained functional at pH 2 (Figure 10B). Since Gal was present in the vicinity of acidic structures in the glycans that were highly associated with binding at pH 2 among the mucin glycans, and there was also a correlation with overall acidic glycans, we hypothesized acidic charge may also confer binding and/or that acidic modification on Galβ3GlcNAc may have beneficial effects on binding. To investigate the first option, we analyzed binding to DNA, which carries a high density of negative charge. *H. suis* did indeed bind DNA at pH 2, demonstrating that negative charge *per se* also confer binding at pH 2 (Figure 10B), but not at pH 7 (Figure 10A).

### *H. suis* binding to mucins can be inhibited by synthetic Galβ3GlcNAcβ3Galβ4Glc and Neu5Acα2,3Galβ3GlcNAcβ3Galβ4Glc

Finally, we demonstrated that binding to pig mucins could be inhibited by pre-treating the bacteria with LNT or sialylated LNT (LS-tetrasaccharide a (LSTa), Neu5Acα2,3Galβ3GlcNAcβ3Galβ4Glc) (p ≤ 0.01, One-way ANOVA, Dunnetts post hoc test) (Figure 10C) Similar to the porcine glycolipids, we identified the presence of type 1 chains in the porcine mucin, where a low molecular structure Galβ3GlcNAcβ3Galβ3GalNAc was identified with a relative abundance of 0–5.5%, while type 2 chains appeared to be the dominant lactosamine units.

## Discussion

**Figure 11.**
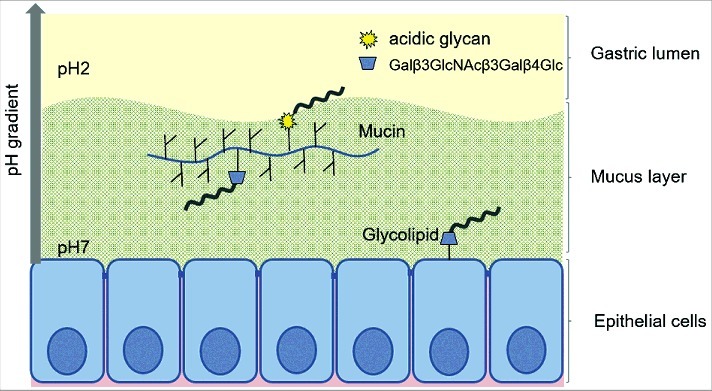
Schematic representation of the two binding modes of *H. suis*. *H. suis* binds to Galβ3GlcNAcβ3Galβ4Glcβ1 at both neutral and acidic pH and to negatively charged glycan structures at acidic pH. Galβ3GlcNAcβ3Galβ4Glcβ1 is present both on glycolipids at the epithelial surface where pH is close to neutral and on secreted mucins in the mucus layer where pH range from neutral to acidic, and *H. suis* binding can thus occur to this structure both when present on glycolipids and on mucins. *H. suis* binding to negatively charged glycan structures at acidic pH is likely to mainly occur to mucin glycans and shed DNA at a distance from the epithelial surface where the pH is acidic. Potentially binding to these structures could also occur after tissue invasion, i.e. at neutral pH when *H. suis* is present in the lamina propria and at acidic pH inside parietal cell canaliculi, however, the distribution of these structures in lamina propria and parietal cells is unknown.

We found *H. suis* in the porcine gastric mucus layer covering the epithelial cells, in lamina propria and in association with acid producing parietal cells. This suggests that the pathogen is exposed to neutral pH when it is present in the lamina propria and mucus close to the epithelial cells and to acidic pH further out towards the gastric lumen as well as when inside parietal cell canaliculi. We characterized the gastric mucins and the mucin glycosylation from *H. suis*-free pigs, with regards to mucin species, solubility and glycosylation, i.e. the environment that *H. suis* meets upon entry to the porcine stomach. We also identified glycolipids present in the porcine stomach, which may confer more intimate *H. suis* adherence than mucins. *H. suis* bound to both mucins and glycolipids. *H. suis* binding to porcine mucins had an acidic pH optimum, differed between individuals and mucin types, and consisted of two binding modes: one that is active only at acidic pH and one that is active at both acidic and neutral pH. *H. suis* bound Galβ3GlcNAcβ3Galβ4Glc on glycoconjugates at both neutral and acidic pH, and to negatively charged structures at acidic pH (visualized in Figure 11), and binding to mucins can be inhibited by Galβ3GlcNAcβ3Galβ4Glc both with and without terminal sialylation.

In line with studies showing that *H. suis* can be present inside canaliculi of the parietal cell [30,31], we found *H. suis* in lamina propria and in association with acid producing parietal cells. The mechanism used by *H. suis* to enter the tissue is currently unknown. The tightly coiled spiral shape and high number of flagella on *H. suis* cells [37] together with the urease activity (that can be directly associated with motility [38) might contribute to the migration of this pathogen into the intracellular canaliculi of gastric parietal cells.

Porcine gastric mucins have previously been demonstrated to be large, oligomeric and highly glycosylated, similarly to human mucins [39,40]. Porcine gastric mucins can be commercially obtained in bulk, however, due to the high prevalence of *H. suis* infection in pigs (60-95%), [1–3] both previous studies and the commercially available mucins are most likely from infected pigs. Here, we characterized the glycosylation of gastric mucins isolated from *H. suis*-free pigs. Out of the tree pigs, two were 8 weeks old and one was 4 months old at the time of euthanization. Age-related glycosylation differences that have been reported between 21d and 180d old pigs [41] were not observed in our samples, and the mucin glycan profile differed to a similar degree between the 8 week old pigs as between the 8 week and 4 months old pigs, suggesting that the detected differences were genetic rather than age-related. Among three studied pigs, we found 109 oligosaccharide structures, whereof only 14 were present in all the examined samples. This indicates high inter-individual variation among porcine gastric mucin glycans, similar to what we previously have shown for human gastric mucins [17]. The higher number of structures found among the human mucin glycans cannot be taken as evidence of higher inter-individual variability in humans than in pigs, as the human samples also include pathological specimens. A major difference between porcine and human gastric mucin glycans is that sulfation, a modification virtually absent among human mucin glycans from healthy stomachs, is a common feature on porcine glycans.

Here we used four methods to analyze *H. suis* binding to glycan structures: Binding of ^35^S-labeled bacteria to glycosphingolipids on thin-layer chromatograms, microtitre based assays using anti-Helicobacter antibody detection or biotinylated bacteria as detection system and binding inhibition assays. Overall, binding was relatively weak compared to the level of adhesion found with *H. pylori*. We did have occasional negative binding results, possibly due to the weak character of the binding, low level of incorporation of radioactive label, or phase variation. The results (both positive and negative binding) shown in this manuscript are only those that were reproduced with at least two methods. We occasionally also detected weak adhesion to other structures (data not shown). These data indicate that *H. suis* also may bind to other structures under certain circumstances. Some of the binding modes found were present at acidic pH, raising a potential concern that the binding may be due to protein denaturing and becoming generally ‘sticky’. However, the binding data is presented after subtracting the background signals of the assay at each pH. Furthermore, the effect of pH on bacterial binding to mucins varied between the mucin samples tested. Among the mucins, there were both human and pig mucins that did not bind *H. suis* (e.g. H2 and P4 SI) at pH 2 and others that displayed strong binding (e.g. H3 and P2 SS). If the binding signal was due to adhesion to denatured protein, the increased signal would be expected to occur to all samples, as the amount of protein core with potential to become denatured is similar between samples. In addition, binding was inhibited by addition of glycan structures, and binding at acidic pH also occurred to DNA, suggesting that binding at acidic pH occurs to charged structures, not to denatured proteins. Both gastric mucins and *H. suis* are adapted to the acidic environment of the stomach, and this may be a reason that protein denaturation did not cause problems in our assays. *H. pylori,* a close relative of *H. suis,* binds to gastric mucins via several modes of adhesion, whereof the BabA and SabA adhesins binding to Le^b^ and SLe^x^ blood group antigens in the mucins have been particularly well characterized [40,42–44]. The BabA and SabA adhesins are absent in the genome of *H. suis*. The porcine and human mucin glycans carry a large range of complex structures, but mucins can carry similar glyco-epitopes as glycolipids, and act as a decoy for the intimate adherence conferred by *Helicobacter* adhesion to glycolipids in the gastric epithelium [21,43,45]. In addition to being the most likely host target that would be beneficial for the pathogen to adhere to, the use of glycolipids simplifies the investigation of the binding specificity, since these compounds have only one glycan, in contrast to the multiple glycans carried by mucins. ^35^S-labeled *H. suis* bound to Galβ3GlcNAcβ3Galβ4Glcβ1Cer. Although low incorporation of radioactive label into this fastidious pathogen made the results difficult to reproduce, we also demonstrated binding to Galβ3GlcNAcβ3Galβ4Glc at both acidic and neutral pH using methods independent of metabolic labelling. In line with the present observation that *H. suis* bound lactotetraosylceramide in pig stomach, *H. pylori* binding to lactotetraosylceramide has been identified in human gastric epithelium, [46] and may thus also function as a *H. suis* human gastric epithelial cell attachment factor. Sulfated lactotetraosylceramide has not been characterized, whereas sialyl-lactotetraosylceramide has to date only been found as an early fetal ganglioside in the brains of young children, [47] and in human meconium [48] and it is not recognized by *H. pylori* [22, 46].

In pigs and humans, structures binding *H. suis* are present both on mucins and glycolipids. Different opinions on the outcome of mucin binding to pathogens exist, where some scientists appear to be of the opinion that binding to mucins gives *H. pylori* a reservoir for re-infection, although we have not seen any experimental data supporting this. We have, however, several lines of support for the function of mucins as decoys for the more intimate adherence to glycolipids. Both in the rhesus monkey model of *H. pylori* infection and in a study of pediatric patients, individuals with mucins with high *H. pylori* binding ability had a lower amount of *H. pylori* in their stomach and lower gastritis than individuals with low binding ability [29,43]. Furthermore, the membrane bound mucin MUC1 can act as a releasable decoy hindering prolonged adherence to epithelial cells *in vitro*, and mice lacking this mucin develop more severe gastritis [45].

The statistical correlation analysis between *H. suis* adhesion to mucins and amount of specific glycan structures implicated two structures to be relevant for binding at neutral pH: Galβ4(S)GlcNAcβ6(Fucα2Galβ3)GalNAcol and GalNAcα3(Fucα2)Galβ3(SGlcNAcβ6)GalNAcol. However, we detected no binding to LNnT (Galβ4GlcNAcβ3Galβ4Glc) conjugated to HSA nor to Galβ4GlcNAcβ3Galβ4GlcNAcβ3Galβ4Glcβ1Cer or GalNAcα3(Fucα2)Galβ3GlcNAcβ3Galβ4Glcβ1Cer, which migrates at a lower level than where we detected *H. suis* binding on the thin layer chromatography. Thus, either other parts of the molecules than these are important for the adhesion or these correlations are not casually related. However, at acidic pH, correlation analysis implicated that sialylation and sulfation were important, a notion that we then demonstrated to be functional both by showing binding to DNA (as a proxy for structurally unrelated negative charge) and by partially inhibiting binding to mucins using a sialylated glycan.

In conclusion, we demonstrated that *H. suis* binding occurs via two modes of adhesion: to Galβ3GlcNAcβ3Galβ4Glcβ1 at both neutral and acidic pH, and to negatively charged structures at acidic pH. Furthermore, binding occurs to both mucins and glycolipids. Since binding glycolipids confer close adherence to host epithelial cells, whereas mucins can act as decoys and hinder access to the epithelial cell surface, the balance between adhesion to mucins versus glycolipids is probably important for the colonization process and the outcome of the disease. Knowledge on the structural basis for these host-pathogen interactions may contribute towards the development of breeding or treatment strategies to prevent diseases.

## Materials and methods

### Samples

#### Porcine samples

The *Helicobacter* infection status of surface and gland mucosa samples collected from the antrum of the stomach from 8 weeks, 4 months and 6 months old pigs originating from different Belgian pig herds (Rattlerow Seghers sow x Piétrain boar), was determined by fluorescence in situ hybridization (FISH, described below) and PCR. DNA was extracted with the DNeasy Tissue Kit (Qiagen, Hilden, Germany) and PCR for *H. suis* was performed as described by De Groote and colleagues. [2] Four pigs were deemed free of *H. suis* infection, and examination of hematoxylin and eosin stained sections from the antrum further confirmed that the *H*. *suis* negative specimens had a normal histological appearance. Mucins were isolated from these 4 pigs not infected with *H. suis* (pig 1 and pig 2 were 8 weeks old, pig 3 was 6 months old and pig 4 was 4 months old). Samples from these pigs, as well as from pigs naturally infected with *H. suis* (collected from slaughterhouses) were used for histology.

#### Human samples

Five samples were selected for this study, based on that they varied highly in mucin glycosylation, [17] thereby providing a wide range of structures for potential differential binding to bacteria. Two samples were from gastric adenocarcinoma tumor (intestinal type) and two were from macroscopically normal mucosa of tumor-affected stomachs, as evaluated by a clinical pathologist. Additionally, one specimen was from the whole gastric wall of a patient who underwent elective surgery for morbid obesity. This patient had no history of peptic ulcer disease, and the stomach was macroscopically normal at the time of operation. The samples with normal histology were obtained  after informed consent and approval of the local ethical committee (Lund University Hospital, Lund, Sweden), whereas the mucins from the tumor samples was from our well characterized mucin library, and these sample were collected in 1983 at the  IMIM-Hospital del Mar, Barcelona, Spain (before the hospital had an ethics committee).

### Immunoflourescence

5 µm thick tissue sections were cut from porcine gastric tissue specimens fixed in Carnoy's solution and embedded in paraffin. Sections were deparaffinized and rehydrated in sequential washes of 95%, 80% and 50% ethyl alcohol, and antigen retrieval was performed using 10 mM sodium citrate (pH 6.0) at 95°C for 10 min. Nonspecific binding was blocked with 1% BSA for 1h. Primary antibody anti-MUC5AC (45M1; Sigma-Aldrich,M5293) was diluted 1:1,000 and incubated at 4°C for 3 h. Sections were washed with cold PBS (140 mM NaCl, 2.7 mM KCl, 10 mM phosphate buffer, pH 7.4) 3 times and incubated with anti-mouse secondary antibody conjugated with Alexa Fluor 350 (Life Technologies, A11045), diluted 1:200 at 4°C for 3 h. Sections were incubated with HCS CellMask™ Red Stain (ThermoFisher, H32712) diluted 1:14,000 at RT for 30 min. Sections were washed again with cold PBS 2 times and with water one time and mounted with ProLong® Gold Antifade Mountant (Life Technologies, P36934).

### FISH

Five μm pig gastric tissue sections from the pigs that mucin samples were isolated from and 7 pigs naturally infected with *H. suis* and fixed in Carnoy's solution were deparaffinized and rehydrated in sequential washes of 95%, 80% and 50% ethyl alcohol. After air drying, a hybridization cocktail containing 40% formamide, 0.1% SDS, 0.9M NaCl, 20mM Tris pH 7.4, nuclease free water and 10 ng/µL fluorescently labeled probes (EUB338 probe: 5′-GCTGCCTCCCGTAGGAGT-3′ and *H. suis* specific probe: 5′-TCTCAGGCCGGATACCCGTCATAGCCT-3′) were added to the sections, and they were placed in a humidified chamber and incubated at 37°C overnight. The slides were washed in wash buffer containing 0.9M NaCl, 25mM Tris pH 7.4 at 50°C for 20 min. They were dipped in room temperature distilled water very briefly, dried at room temperature and mounted with ProLong® Gold Antifade Mountant containing DAPI (Life technologies, P36935).

### Isolation of gastric mucins

#### From pigs

Mucins were isolated from the antrum of 4 pigs without *H. suis* infection. Tissues were stored at -80°C after excision. The frozen tissue pieces were drenched in 10 mM sodium phosphate buffer (pH 6.5) containing 0.1 mM phenylmethyl sulphonyl fluorid (PMSF). The surface and gland mucosa of the antrum of the pig stomachs was removed by scraping the tissue with a glass microscope slide. The scrapings were placed into five volumes of extraction buffer (6 M GuHCl, 5 mM EDTA, 10 mM sodium phosphate buffer, pH 6.5) containing 0.1 M PMSF and were dispersed with a Dounce homogenizer (four strokes with a loose pestle) and stirred slowly at 4°C overnight. The insoluble material was removed by centrifugation at 23,000 × g for 50 min at 4°C (Beckman JA-30 rotor). The supernatant corresponded to the GuHCl soluble mucins and the pellet was re-extracted twice with 10 mL extraction buffer. The final extraction residue was reduced with 10 mL of 10 mM 1,4-dithiothreitol (DTT) in reduction buffer (6 M GuHCl, 5 mM EDTA, 0.1 M Tris-HCl buffer, pH 8.0) for 5 h at 37°C followed by alkylation with 25mM iodoacetamide overnight in the dark at 23–24°C. After centrifugation at 23,000 × g for 50 min at 4°C (Beckman JA-30 rotor), the supernatant contained the GuHCl insoluble mucins. The insoluble and soluble samples were dialyzed against 10 volumes of extraction buffer for 8 h and 24 h, respectively. The samples were adjusted to 26 mL with extraction buffer. CsCl was added to the samples to give 1.39 g/mL starting density by gentle stirring. The samples were transferred into Quick Seal ultracentrifuge tubes and centrifuged at 40,000 × g for 90h at 15°C. The fractions were collected from the bottom of the tubes with a fraction collector equipped with a drop counter. After this procedure, the mucins were largely separated from the non-mucin low glycosylated proteins (as determined in combination with the carbohydrate detection method below), however, in some samples there was a partial overlap between the DNA peak and the mucins, and thus, all mucins were further purified by pooling and diluting the mucin containing fractions in CsCl/0.5 M GuHCl and repeating the ultracentrifugation, as described above, with a starting density at 1.50 g/mL. Again, the fractions were analyzed for density using a Carlsberg pipette as a pycnometer and for DNA content by measuring absorbance at 260 nm for carbohydrate content (below) and for MUC5AC using enzyme-linked immunosorbent assay (ELISA) (below).

#### From humans

Rinsed and frozen tissue pieces were drenched in 10 mM sodium phosphate buffer (pH 6.5) containing 0.1 mM PMSF. The surface and sub-mucosa of normal tissue was removed by scraping the tissue with a glass microscope slide. Tumor tissue was homogenized without prior scraping. The material was immersed in liquid nitrogen and pulverized in a Retch tissue pulverizor (Retch GmBH & Co., Haan, Germany), then placed into five volumes of extraction buffer containing 0.1 mM PMSF and dispersed using a Dounce homogenizer (four strokes with a loose pestle) and stirred slowly at 4°C overnight. The insoluble material was removed by centrifugation at 23,000 × g for 50 min at 4°C (Beckman JA-30 rotor). In this study, we only used mucins present in the supernatant, where most of the human MUC5AC and MUC6 molecules usually are found. Supernatants were dialyzed against ten volumes of extraction buffer for 24 h and then filled up to 26 mL with extraction buffer. Cesium chloride was added to the samples to give 1.39 g/mL starting density by gentle stirring. The samples were centrifuged at 40,000 × g for 90 h at 15°C. Analytical assays on mucin fractions were performed as for the pig mucins.

### Carbohydrate detection

Gradient fractions were diluted in 0.5 M GuHCl and coated on to Nunc 96-well polisorp plates (ThermoFisher Scientific, 475094) overnight at 4°C. The plates were washed 3 times with DELFIA washing solution (5 mM Tris-HCl, 0.15 M NaCl, 0.005% Tween 20, 0.01% NaN_3_, pH 7.75). Periodate-oxidation was performed for 20 min with 25 mM sodium metaperiodate in 0.1 M sodium acetate buffer (pH 5.5). The plates were washed again 3 times with DELFIA washing solution and one time with PBS/0.05% Tween, and the wells were blocked for 1h with DELFIA blocking solution (50 mM Tris-HCl, 0.15 M NaCl, 90 µM CaCl_2_, 4 µM EDTA, 0.02% NaN_3_, and 0.1% BSA) at 22–24°C. After discarding the blocking buffer, the plates were incubated for 1 hour with 2.5 µM biotin-hydrazide solution in 0.1 M sodium acetate buffer (pH 5.5) at 22–24°C. The plates were washed again 3 times and then incubated for 1 hour at 22–24°C with europium labelled Streptavidin (PerkinElmer, 1244–360) 1:1,000 in DELFIA assay buffer (PerkinElmer, 1244-111). This was followed by washing the plates six times with DELFIA washing solution and incubated for 5 min on a shaker with DELFIA enhancement buffer (PerkinElmer, 1244-114). The fluorescence was measured using a Wallac 1420 VICTOR2 plate reader with Europium label protocol (PerkinElmer, Waltham, MA, USA).

### ELISA for MUC5AC detection

Mucin samples were diluted in 0.5 M GuHCl and coated onto Nunc 96-well polisorp plates (ThermoFisher Scientific, Roskilde, Denmark) overnight at 4°C. The plates were washed three times with PBS/0.05% Tween with ELISA washer. The wells were blocked with 1% blocking reagent for ELISA (Roche, 11112589001) containing 0.05% Tween 20 at 22–24°C for 1 hour. After discarding the blocking buffer, the wells were incubated with anti-MUC5AC primary antibody (45M1, Sigma-Aldrich, M5293) diluted 1:8,000 in blocking buffer at 22–24°C for 1 hour, followed by washing with washing buffer (PBS/0.05% Tween 20). The wells were incubated with horseradish peroxidase (HRP) conjugated anti-mouse IgG secondary antibody diluted in blocking buffer at 22–24°C for 1 hour and then washed with washing buffer. 3,3′,5,5′-Tetramethylbenzidine (TMB) substrate for ELISA (Sigma-Aldrich, T0440) was added to the wells and the color development was monitored. The reaction was stopped after 20 minutes with 0.5 M H_2_SO_4_ and absorbance was measured at 450 nm.

### Proteomic analysis of gastric mucins

Mucin samples (60 μg) were reduced (10mM DTT, 70°C for 1 h) and alkylated (25 mM iodoacetamide (IAA) for 30 min at room temperature, in the dark). The DTT and IAA were removed using a spin filter with Amicon Ultra 30kDa cut-off (Merck Millipore, Billerica, MA). The filter was pre-washed twice with 200 μL 0.1 M NaOH, followed by 200 μL H_2_O. Samples were added and washed thrice with 200 μL 50 mM (NH_4_)HCO_3_ by centrifugation (5 min at 13,200 rpm). The filter was then placed up-side down in the new tube and the proteins were eluted with 2 × 50 μL (NH_4_)HCO_3_. Enzymatic digestion was performed with trypsin (1:100 enzyme to protein, V511A, Promega, Madison, WI) and 50 mM (NH_4_)HCO_3_ and incubated at 37°C for 16 h. C18 ZipTips (Millipore) were used to enrich the tryptic peptides according to the manufacturer's instructions before proteomic analysis.

Pre- and analytical columns were packed in-house with 3 μm C18 particles (Dalco Chromtech, Stockholm, Sweden) with 4 cm x 75 μm I.D. and 15 cm x 50 μm I.D., respectively. Solvent A was 0.2% formic acid and solvent B was 0.2% formic acid in 80% acetonitrile. A linear gradient was set as follows: 0% B for 5 min, then a gradient up to 35% B in 70 min and to 80% B in 5 min. A 20 min wash at 80% was used to ensure that the sample was eluted from the column and to prevent carryover, and a 25 min equilibration with 100% completed the gradient. The column was attached to an Agilent 1100 series HPLC (Agilent Technologies, Santa Clara, CA) and then samples were analyzed by MS, on an LTQ-Orbitrap XL (Thermo Scientific), in positive ion mode for MS and MS^2^ analysis. The spray voltage was set to 2 kV, and the ion transfer tube was set to 200 °C. The full scans were acquired in a Fourier transform MS mass analyzer that covered *m/z* range of 400–2000. The MS^2^ analysis was performed under data-dependent mode to fragment the top five precursors using collision-induced dissociation (CID). For CID, a normalized collision energy of -35 eV, an isolation width of *m/z* 1.0, an activation Q value of 0.250, and a time of 30 ms were used. Peptide MS/MS spectra were searched against UniProt and NCBI pig protein databases using Mascot server (v.2.2.04, Matrix Science Inc., MA, USA) with in-house build mucin database. The amino acid sequences of the *Sus scrofa* MUC5AC and MUC5B homologs were previously only partial [49]. They were improved here to cover a larger proportion of the full-length sequences using various sources of information. We used 1) available partial protein and mRNA sequences of Genbank and UniProt, 2) in house sequencing of selected portions of the mRNAs and 3) analysis of the identity of VWD domains using described methods [50]. Our assembly of the pig sequences was also aided by available sequences of bovine Muc5ac and Muc5b. We also used a method to identify shorter peptides of the two mucins where tblastn [49] was used to search *Sus scrofa* RNA-Seq data, using bovine mucins as queries. The final sequences of *S. scrofa* Muc5ac and Muc5b used in this project are not entirely complete and are not guaranteed to be correct in every amino acid position, but provided nevertheless us the ability to efficiently discriminate between MUC5AC and MUC5B. For *S. scrofa* MUC2 and MUC6, we used protein sequences available in Genbank, XP_013845199.1 (MUC2) and XP_013845183.1 (MUC6). Both of these entries seem to comprise near full-length mucin sequences. Only peptides with a mass deviation lower than 10 ppm were accepted and at least two peptide sequences with manual inspection were used for positive protein identification.

### Release of *O*-linked mucin oligosaccharides

Sample preparation for LC-MS/MS analysis of glycans: purified porcine gastric mucins were dot-blotted onto polyvinylidene fluoride (PVDF) membrane (Immobilin P, Millipore, Billerica, MA, United States) (approx. 20 μg of protein per spot, two spots per sample), stained with Alcian Blue 8GX Alcian Blue 8GX (A5268, Sigma-Aldrich) for 1hr at RT and destained with methanol. The *O*-glycans were released from PVDF membranes by incubation with 50 μL 0.5 M NaBH_4_ in 50 mM NaOH, 16 hr, at 50°C [51]. The reductive β-elimination reaction was quenched by addition of 1 μL of acetic acid and the samples were desalted with 35 μL of AG50WX8 cation exchange slurry (Bio-Rad, Hercules, CA) packed on the top of C18 zip tips (Millipore). The samples were then dried in SpeedVac and borate complexes were removed by repeated addition/evaporation of 50 μL of methanol (five times). The released oligosaccharides were dissolved in water.

Selected structures were also analyzed with an LC-LTQ Orbitrap mass spectrometer (Thermo Electron Corp.).The LC setup was the same as that described for LC-ESI-MS/MS. Ions isolated for HCD fragmentation were acquired with a resolution of 7,500 and subjected to collision with the collision energy set to 95% with an activation time of 30 ms. The data were processed using the Xcalibur software (version 2.2, Thermo Scientific) and manually interpreted from their MS/MS spectra.

### *Helicobacter suis* culture conditions

*Helicobacter suis* strain HS1 and HS5 were cultured in biphasic Brucella culture plates (BD BBL, 211086) containing 20% fetal bovine serum (FBS) (HyClone, SH30071.01), Vitox supplement (Oxoid, SR0090H), Skirrow *Campylobacter* selective supplement (Oxoid, SR0069E), and 5 mg/L amphotericin B. The pH of the agar was adjusted to 5. One mL sterile brucella broth (pH 5) was added onto the agar surface. The bacteria were cultured under microaerobic conditions at 37°C [37].

### Binding to purified mucins and glycoconjugates using antibody detection

Mucin samples were diluted in 0.5 M GuHCl and glycoconjugates of Le^b^, SLe^x^, LacdiNAc and Lacto-N-tetraose were diluted in PBS to 4 µg/mL concentrations and coated on 96-well polysorp plates overnight at 4°C. The plates were washed three times with washing buffer (PBS with 0.05% Tween 20) and the wells were blocked for 1 hour with 1% Blocking Reagent for ELISA (Roche, 11112589001). After discarding the blocking buffer, bacteria were diluted to an OD_600_ of 0.05 in blocking buffer (pH 2–7) containing 10mM citric acid and 0.05% Tween 20. For the binding inhibition assay, *H. suis* was pre-incubated with 0.5mg/mL of one of the following sugars: Lacto-N-tetraose (LNT), LS-tetrasaccharide a (LSTa) (OligoTech, GLY010, GLY081), D-glucose, D-galactose, N-acetyl-glucosamine, L-fucose, N-acetyl-galactosamine for 30 min at RT prior to the incubation with mucins. The pH of the blocking buffer was adjusted with the addition of HCl that between pH 2 and pH 7 changed the osmolarity of the buffer with approximately 0.25%. The 0.25% change was determined to have negligible effects on the experiments based on that pilot experiments that included addition of 10 mM citrate, which increased the osmolality with approximately 9% gave results very similar to those presented herein. The bacterial suspension was added to the plates, which then were incubated for 2 hours at 37°C and 120 rpm. The plates were washed three times with washing buffer and then incubated for 1 hour at 22–24°C with rabbit anti-*H. pylori* serum diluted 1:1,000 in blocking buffer. The plates were washed three times and incubated for 1 hour at 22–24°C with horseradish peroxidase conjugated anti-rabbit IgG diluted 1:10,000 in blocking buffer. After further washing steps, TMB substrate was added to the wells and the plates were incubated for 20 min at 22–24°C. The reaction was stopped with an equivalent amount of 0.5 M H_2_SO_4_ and absorbance was measured in a microplate reader at 450 nm. The data are presented after subtracting the background signal (the binding of bacteria to the plastic wells and the binding of antibody to the mucin). Analyses presented in all figures except 10A was performed this way whereas analyses presented in 10A was performed using biotinylated *H. suis* (as described [45) due to high background signal from the antibody detection system to some HSA-glycoconjugates.

### Binding to purified mucins and glycoconjugates using biotinylated bacteria

Biotinylation of bacteria was performed as previously described [24]. Mucin samples were diluted in 0.5 M GuHCl to 4 µg/mL and coated on 96-well polysorb plates over night at 4°C. The plates were washed three times with washing buffer (PBS with 0.05% Tween 20) and the wells were blocked for 1 hour with 1% Blocking Reagent for ELISA (Roche, 11112589001). After discarding the blocking buffer, biotinylated bacteria were diluted to an OD_600_ of 0.05 in blocking buffer (pH 2–7). The bacterial suspension was added to the plates, which then were incubated for 2 hours at 37°C and 120 rpm. The plates were washed 3 times and then incubated for 1 hour at 22–24°C with blocking buffer containing 1 µg/mL of horse radish peroxidase conjugated streptavidin. After further washing steps, TMB substrate was added and the plates were incubated for 5 min. The reaction was stopped with an equivalent amount of 0.5 M H_2_SO_4_ and the plates were read in a microplate reader at 450 nm after color stabilization.

### Isolation of glycosphingolipids from pig stomach

Total acid and non-acid glycosphingolipids were isolated from the stomachs (corpus and fundus region) of four *H. suis* negative pigs, as previously described [46].

### Binding of H. suis and H. pylori to glycosphingolipids on thin-layer chromatograms

The glycosphingolipids (40 µg/lane) were separated on aluminum-backed silica gel plates, using chloroform/methanol/water 60:35:8 (by volume) as solvent system. Metabolic labeling of *H. suis* strain HS5 and *H. pylori* strain J99 was done by culture in the presence of ^35^S-methionine as outlined in [46]. Binding of ^35^S-labeled bacteria to glycosphingolipids on thin-layer chromatograms was done as described before [46]. Bound bacteria were detected by autoradiography (12 h).

### Endoglycoceramidase digestion

Endoglycoceramidase II from *Rhodococcus* spp. (Takara Bio Europe S.A., Gennevilliers, France) was used for hydrolysis of the non-acid glycosphingolipids. The glycosphingolipids (50 μg) were resuspended in 100 μL 0.05 M sodium acetate buffer, pH 5.0, containing 120 μg sodium cholate, and sonicated briefly. Thereafter, 1 mU of enzyme was added, and the mixture was incubated at 37 °C for 48 h. The reaction was stopped by addition of chloroform/methanol/water to the final proportions 8:4:3 (by volume). The oligosaccharide-containing upper phase thus obtained was separated from detergent on a Sep-Pak QMA cartridge (Waters). The eluent containing the oligosaccharides was dried under nitrogen.

### LC-ESI MS-analysis of mucin- and glycosphingolipid-derived oligosaccharides

Oligosaccharides were resuspended in 50 μL of water and analyzed by LC-ESI/MS as described [35]. The oligosaccharides were separated on a column (100 × 0.250 mm) packed in-house with 5 μm porous graphite particles (Hypercarb, Thermo-Hypersil, Runcorn, UK). An autosampler, HTC-PAL (CTC Analytics AG, Zwingen, Switzerland) equipped with a cheminert valve (0.25 mm bore) and a 2 μL loop, was used for sample injection. An Agilent 1100 binary pump (Agilent technologies, Palo Alto, CA) delivered a flow of 250 μl/min, which was split down in an 1/16” microvolume-T (0.15 mm bore) (Vici AG International, Schenkon, Switzerland) by a 50 cm x 50 μm i.d. fused silica capillary before the injector of the autosampler, allowing approximately 2–3 μl/min through the column. The oligosaccharides (3 μL) were injected on to the column and eluted with an acetonitrile gradient (A: 10 mM ammonium bicarbonate; B: 10 mM ammonium bicarbonate in 80% acetonitrile). The gradient (0-50% B) was eluted for 46 min, followed by a wash step with 100% B, and equilibration of the column for 24 min. A 30 cm x 50 μm i.d. fused silica capillary was used as transfer line to the ion source.

The saccharides were analyzed in negative ion mode on an LTQ linear ion trap mass spectrometer (Thermo Electron, San José, CA). The IonMax standard ESI source on the LTQ mass spectrometer was equipped with a stainless steel needle kept at −3.2 kV. Compressed air was used as nebulizer gas. The heated capillary was kept at 310 °C, and the capillary voltage was −50 kV. Full-scan (*m/z* 380–2,000, 2 microscans, maximum 100 ms, target value of 30,000) was performed, followed by data dependent MS^2^ scans of the three most abundant ions in each scan (2 microscans, maximum 100 ms, target value of 10,000). The threshold for MS^2^ was set to 300 counts. Normalized collision energy was 35%, and an isolation window of 3 u, an activation q = 0.25, and an activation time of 30 ms, was used. Data acquisition and processing were conducted with Xcalibur software (Version 2.0.7 or 2.2). Selected structures were also analyzed with an LC-LTQ Orbitrap mass spectrometer (Thermo Electron Corp.).The LC setup was the same as that described for LC-ESI-MS/MS. Ions isolated for HCD fragmentation were acquired with a resolution of 7,500 and subjected to collision with the collision energy set to 95% with an activation time of 30 ms.

Manual assignment of glycan sequences was done on the basis of knowledge of mammalian biosynthetic pathways, with the assistance of the Glycoworkbench tool (Version 2.1), and by comparison of retention times and MS^2^ spectra of oligosaccharides from reference glycosphingolipids [35].

## Supplementary Material

1460979.zip

## Abreviations

*BabA*: Blood group antigen binding adhesin
*CID*: Collision-induced dissociation
*DTT*: Dithiothreitol
*EIC*: Extracted ion chromatogram
*ELISA*: Enzyme-linked immunosorbent assay
*FBS*: Fetal bovine serum
*GI*: Gland insoluble
*GS*: Gland soluble
*GuHCl*: Guanidinium chloride
*HCD*: Higher-energy collisional dissociation
*HpaA*: *Helicobacter pylori* adhesin A
**H. pylori**: *Helicobacter pylori*
**H. suis**: *Helicobacter suis*
*HRP*: Horseradish peroxidase
*IAA*: Iodoacetamide
*Le*: Lewis
**LNnT**: Lacto-N-neotetraose
*LNT*: Lacto-N-tetraose
*LSTa*: LS-tetrasaccharide a
*MALT*: Mucosa-associated lymphoid tissue
*OD*: Optical density
*OMPs*: Outer membrane proteins
*PMSF*: Phenylmethyl sulphonyl fluorid
*PVDF*: Polyvinylidene fluoride
*SabA*: Sialic acid binding adhesin
*SI*: Surface insoluble
*SLe^a^*: Sialyl-Le^a^
*SLe^x^*: Sialyl-Le^x^
*SS*: Surface soluble
*TMB*: 3,3′,5,5′-Tetramethylbenzidine
